# A randomized longitudinal dietary intervention study during pregnancy: effects on fish intake, phospholipids, and body composition

**DOI:** 10.1186/1475-2891-14-1

**Published:** 2015-01-02

**Authors:** Marja Bosaeus, Aysha Hussain, Therese Karlsson, Louise Andersson, Lena Hulthén, Cecilia Svelander, Ann-Sofie Sandberg, Ingrid Larsson, Lars Ellegård, Agneta Holmäng

**Affiliations:** Department of Physiology, Institute of Neuroscience and Physiology, Sahlgrenska Academy at University of Gothenburg, Gothenburg, Sweden; Department of Internal Medicine and Clinical Nutrition, Institute of Medicine, Sahlgrenska Academy at University of Gothenburg, Gothenburg, Sweden; Division of Life Sciences/Food Science, Department of Chemical and Biological Engineering, Chalmers University of Technology, Gothenburg, Sweden; Department of Endocrinology, Diabetology and Metabolism, Sahlgrenska University Hospital, Gothenburg, Sweden

**Keywords:** Pregnancy, Fish intake, Meat intake, Body composition, Fatty acids

## Abstract

**Background:**

Fish and meat intake may affect gestational weight gain, body composition and serum fatty acids. We aimed to determine whether a longitudinal dietary intervention during pregnancy could increase fish intake, affect serum phospholipid fatty acids, gestational weight gain and body composition changes during pregnancy in women of normal weight participating in the Pregnancy Obesity Nutrition and Child Health study. A second aim was to study possible effects in early pregnancy of fish intake and meat intake, respectively, on serum phospholipid fatty acids, gestational weight gain, and body composition changes during pregnancy.

**Methods:**

In this prospective, randomized controlled study, women were allocated to a control group or to a dietary counseling group that focused on increasing fish intake. Fat mass and fat-free mass were measured by air-displacement plethysmography. Reported intake of fish and meat was collected from a baseline population and from a subgroup of women who participated in each trimester of their pregnancies. Serum levels of phospholipid arachidonic acid (s-ARA), eicosapentaenoic acid (s-EPA), and docosahexaenoic acid (s-DHA) were measured during each trimester.

**Results:**

Weekly fish intake increased only in the intervention group (n = 18) from the first to the second trimester (median difference 113 g, p = 0.03) and from the first to the third trimester (median difference 75 g, p = 0.01). In the first trimester, fish intake correlated with s-EPA (r = 0.36, p = 0.002, n = 69) and s-DHA (r = 0.34, p = 0.005, n = 69), and meat intake correlated with s-ARA (r = 0.28, p = 0.02, n = 69). Fat-free mass gain correlated with reported meat intake in the first trimester (r = 0.39, p = 0.01, n = 45).

**Conclusions:**

Dietary counseling throughout pregnancy could help women increase their fish intake. Intake of meat in early pregnancy may increase the gain in fat-free mass during pregnancy.

## Background

Human pregnancy involves large physiological changes, including increases in plasma volume and extracellular fluids and production of amniotic fluid, growth of fetus, mammary glands, uterus and placenta, and deposition of fat mass (FM). Thus, both FM and fat-free mass (FFM) increase. Although a healthy gestational weight gain (GWG) is needed to meet the needs of the fetus and the neonate, excessive increases in maternal weight are detrimental for both mother and child. According to a model in one study, GWG in well-nourished women is 13.8 kg, including 4.3 kg of fat deposition [1]. However, GWG shows large interindividual variation, and average GWG is lower at higher body mass index (BMI) [2, 3].

Reaching optimal GWG is complex, taking into account the health of both mother and child. New GWG recommendations were proposed in 2009 [4], in which a weight gain of 11–16 kg was recommended for normal weight women. In fact, even in women of normal weight, large GWG is associated with increased risk of cesarean section, preeclampsia [2], high birth weight (>4500 g) [5, 6], babies born large for gestational age [2], more frequent complications in pregnancy and delivery [7], and more weight retention postpartum [8]. On the other hand, low GWG in normal weight women increases the risk of giving birth to babies that are small for gestational age [2] or <3000 g [3] and is associated with shorter gestation [8]. Since GWG is different from an adverse adipose tissue hyperplasia, body composition should be investigated.

Body composition may be affected by intake of long chain (LC) n-3 polyunsaturated fatty acid (PUFA), resulting in decreased FM [9]. During pregnancy, fatty acids are important for the fetal development. LC n-3 fatty acids are essential for this process [10]. For example, docosahexaenoic acid (DHA) is an important component of neural and retinal membranes and accumulates in the brain during gestation and the postnatal period [10]. Together with eicosapentaenoic acid (EPA), DHA is rapidly taken up by the fetal brain during gestation and in the first years of life [11]. Intake of PUFAs during pregnancy is associated with a lower frequency of preterm birth and a lower risk of intrauterine growth restriction and pregnancy induced hypertension [11].

A major contributor of LC n-3 PUFA in the Swedish diet is fish [12]. Fish is a major source of EPA and DHA [13] and an important source of vitamin D and selenium, but is a small contributor of calories [12]. Meat is an important contributor of protein in the diet. Meat contains several nutrients (vitamin D, iron, and zinc). Red meat also contains saturated fat and n-6 fatty acids such as arachidonic acid (ARA), the latter being precursor to pro-inflammatory eicosanoids [14].

Much research in gestational nutrition has focused on supplements of different nutrients. Nevertheless, to achieve healthy weight gain and fetal development during pregnancy, emphasis should be put on the entire diet. Thus, general recommendations should include dietary advice on suitable food items rather than on dietary supplements.

In this study, we sought to determine whether a longitudinal dietary intervention during pregnancy could increase fish intake, and affect serum phospholipid fatty acids, gestational weight gain, and body composition changes during pregnancy in normal weight women. A second aim was to study possible effects in early pregnancy of fish intake and meat intake, respectively, on serum phospholipid fatty acids, gestational weight gain, and body composition changes.

## Methods

### Study design and participants

**Figure 1:**
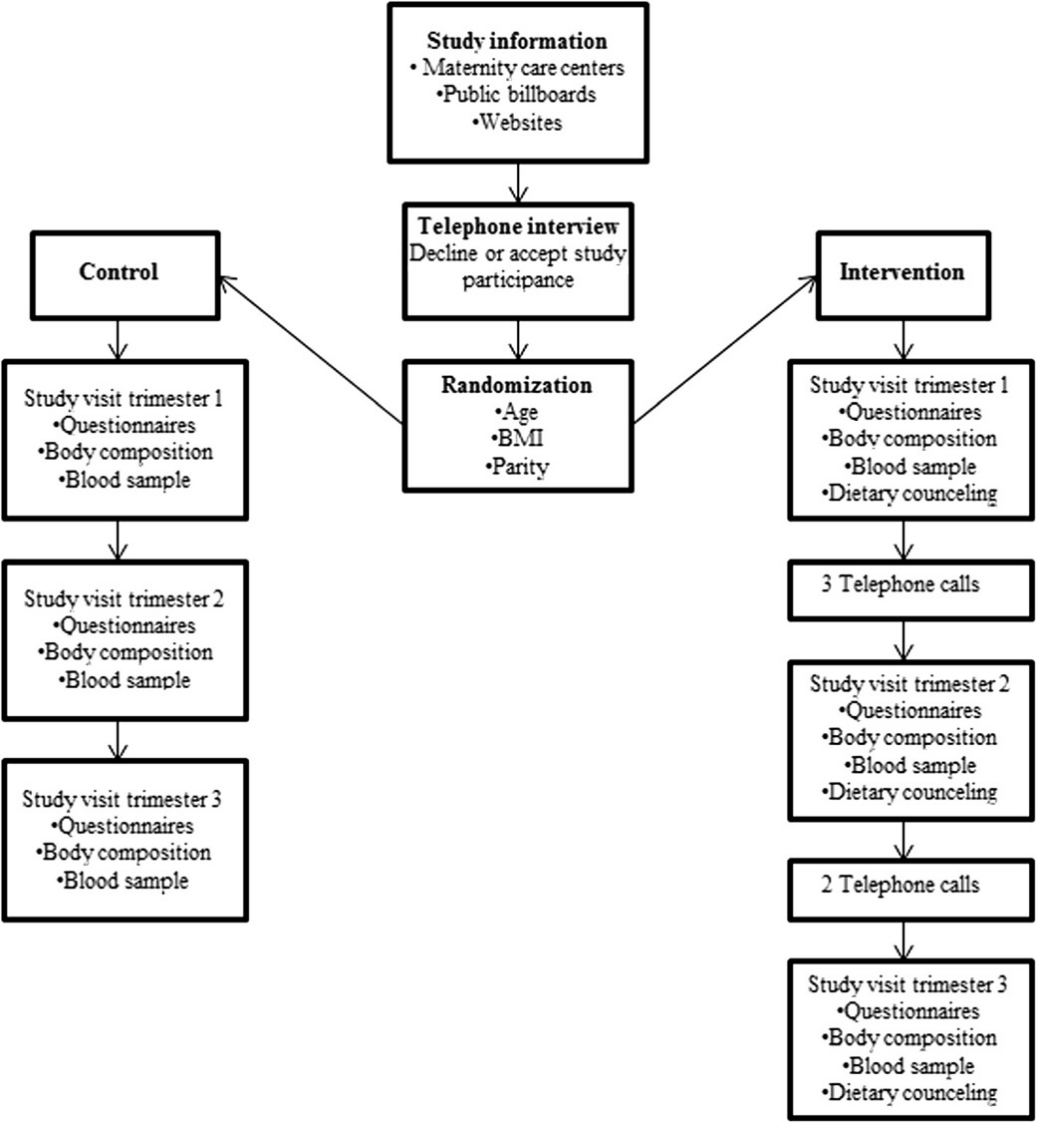
**Pregnancy obesity nutrition and child health study protocol.** Recruitment process, randomization and study visit flow in the control and intervention groups. Trimester 1, pregnancy weeks 8–12; trimester 2, pregnancy weeks 24–26; trimester 3, pregnancy weeks 35–37.

Between April 2009 and December 2012, normal weight pregnant women (n = 101) were recruited for the Pregnancy Obesity Nutrition and Child Health study (PONCH). PONCH is a longitudinal randomized dietary intervention study in pregnant Swedish women of normal weight (BMI 18.5-24.9 kg/m^2^). The PONCH study was approved by the local ethics committee at the University of Gothenburg (nr 402–08) and is performed at the Sahlgrenska University Hospital, Gothenburg, Sweden. Inclusion criteria were age 20–45 years and self-reported BMI (based on weight and height) of 18.5-24.9 kg/m^2^ at the time of recruitment. Self-reported BMI was only used for inclusion. Exclusion criteria were non-European descent, self-reported diabetes, use of neuroleptic drugs, and vegetarianism or veganism. Women who entered the study but had a miscarriage, abortion, intrauterine fetal death, sudden infant death syndrome, duplex pregnancy, or delivery before pregnancy week 34 were excluded from this analysis. Women were recruited through oral and written information given at maternity care centers in Gothenburg, postings at public billboards, and advertisement on a website for pregnant women (Figure 1). All women were living in the Västra Götaland region of Sweden. All women received oral and written information and signed an informed consent before entering the study. After agreeing to participate in the study, women were randomized to a control group or an intervention group. Randomization was done by a computerized program, developed at the department and subjects were matched for age, BMI and parity. Women were included and the first study visit took place during the first trimester (pregnancy weeks 8–12). Follow-ups were done in the second trimester (pregnancy weeks 24–26) and the third trimester (pregnancy weeks 35–37) (Figure 1). Briefly, the study visits took place at the Sahlgrenska University Hospital and included collection of blood samples and body composition measurements. Also, women filled in questionnaires in connection with study visits. Women in the intervention group received dietary counseling, as described below. Women were instructed to fast over-night before study visits.

**Figure 2:**
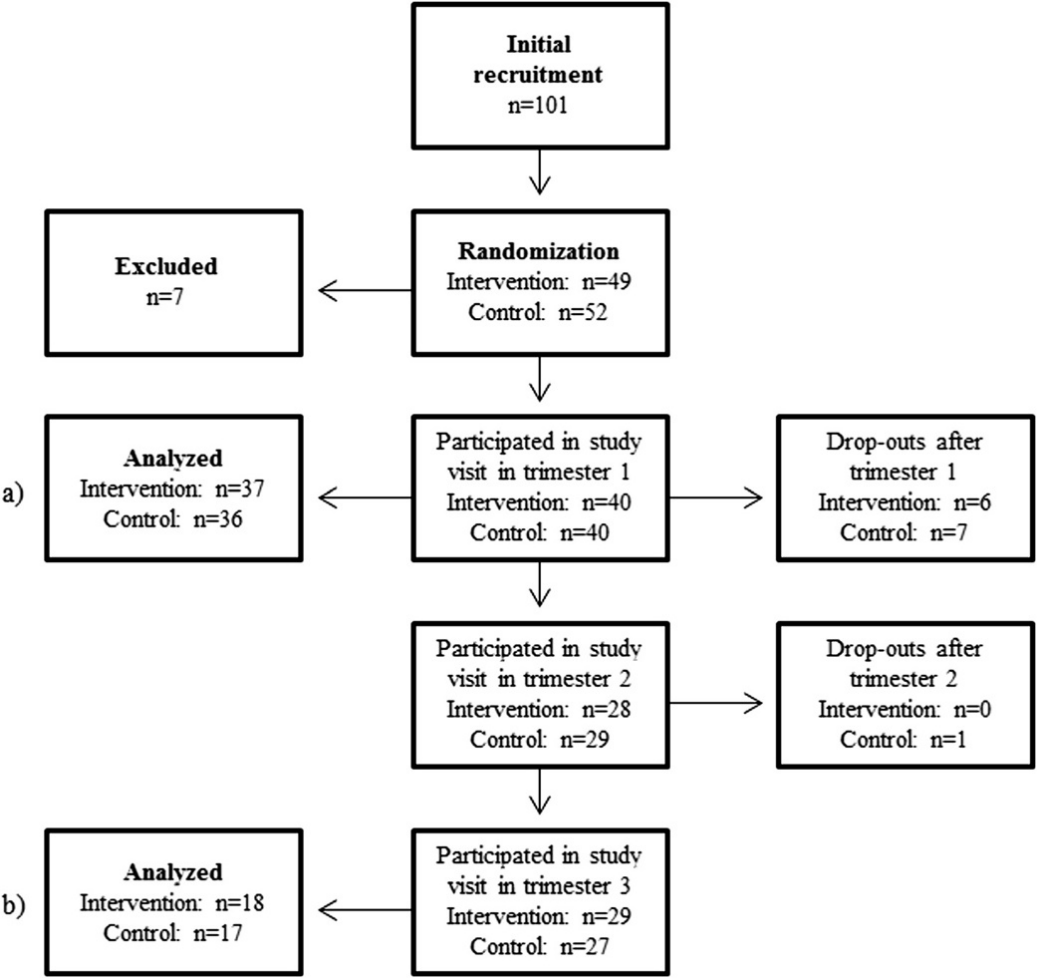
**Recruitment, randomization, participation, drop-outs, exclusion and final data analysis groups of normal weight women in the pregnancy obesity nutrition and child health study.** Flow chart illustrating the number of normal weight women that were recruited, were randomized to intervention or control groups, participated in study visits, and were drop-outs. Women were excluded for miscarriage after trimester 1 (n = 2), intrauterine fetal death (n = 1), duplex pregnancy (n = 1), sudden infant death syndrome (n = 1), abortion (n = 1), and delivery before pregnancy week 34 (n = 1). For data analyses, we defined two populations: a) a pooled “early pregnancy” population at baseline and b) a subpopulation of all women who participated in all 3 trimesters.

In summary, the data were analyzed for one baseline population that participated in the first trimester (“early pregnancy”), where women in the intervention and control groups were pooled (Figure 2). Subgroup analyses were then conducted for women who had measurements of fish intake, body composition, and serum phospholipid fatty acids in all three trimesters (Figure 2; see Results).

### Questionnaires and dietary assessment

A self-administered dietary questionnaire was used to assess energy intake during the three previous months [15]. The questionnaire has a semiquantitative food frequency design. It has been validated in Swedish men and nonpregnant women against a 4-day food record and 24-h energy expenditure and nitrogen excretion. From these comparisons, valid estimates of energy intake were obtained in normal weight and obese subjects [15]. In addition, participants completed a food frequency questionnaire developed at this department to ascertain their weekly intake of fish and meat. We analyzed fish/shellfish and meat intake from the number of hot meals per week containing these food items. The frequency reported was then converted into grams by assuming that a serving of fish was equal to 150 g and a serving of meat equal to 175 g, based on serving sizes recommended by the Norwegian Health Authorities [16].

### Dietary intervention

Dietary counseling was provided by registered dieticians. The aim was to increase adherence to the dietary recommendations for pregnant women stated by the Swedish National Food Agency [17] and in the Nordic Nutrition Recommendations (NNR) 2004 [18]. Participants received dietary counseling on the day of their visit to Sahlgrenska University Hospital for the other measurements in the PONCH study. Participants in the intervention group were advised to consume three meals of fish per week, and advice was also given on the types of fish to consume to avoid pollutants. Additionally, women were advised to generally lower sugar intake in order to reach to <10 E%. Furthermore, women were advised to eat 500 g of vegetables and fruits per day, and finally to increase daily energy intake by 350 kcal in the second trimester and by 500 kcal in the third trimester. Advice on suitable amounts and choices of vegetables and fruits and appropriate snacks was given. Additionally, diet quality was individually adjusted as needed, and counseling was also given on fat quality, food frequency, fibre intake, and nutrient density according to the NNR [18]. After the first visit, women were repeatedly called by telephone (three times between the study visits in the first and the second trimesters and twice between the study visits in the second and third trimesters) to remind them of the recommendations and were followed-up during study visits in the second and third trimesters.

### Anthropometrics and body composition

Body composition was measured by air-displacement plethysmography, performed with the Bod Pod Gold Standard system (Bod Pod 2007 A, Life Measurement, Concord, CA) and software versions 4.2.1 and 5.2.0. Subjects were weighed dressed in bathing cap and underwear after fasting overnight. Height was measured to the nearest 0.5 cm. BMI was calculated by the Bod Pod software, using height to the nearest whole centimeter and measured body weight (BW). Software quality checks and scale calibrations were routinely performed. The Bod Pod system measured BW with a modified Tanita BWB-627-A electronic scale. Body volume was measured twice in the Bod Pod. The Bod Pod system asked for a third measurement if the first two measurements were inconsistent; the criteria for consistency were not specified by the manufacturer. Predicted lung gas volume was used to calculate body composition, and body fat percent was calculated with the Siri equation [19], assuming FM density of 0.9000 kg/L and FFM density of 1.1000 kg/L. Fat% was calculated as ((4.95/body density – 4.50)*100). FM (kg) was calculated as (Fat%*BW/100). FFM (kg) was calculated as (BW-FM). Birth weights were collected from midwives’ records.

### Laboratory analyses

Venous blood samples were obtained after an overnight fast. Biochemical analyses were performed by the accredited (SWEDAC ISO 15189) Laboratory for Clinical Chemistry, Sahlgrenska University Hospital. ElektroChemiLuminiscenseImumunoAssay sandwich-method on a Cobas 6000 with Insulin Elecsys (Roche Diagnostica Scandinavia AB) was used for the serum insulin analysis. The insulin assay had a coefficient of variation of 10% at 6, 20, and 180 mU/liter. Quantitative insulin sensitivity check index (QUICKI) was calculated as 1/(Ln(s-insulin)(mU/L) + Ln(p-glucose)(mmol/L)) [20].

### Analyses of long chain polyunsaturated fatty acids in serum phospholipids

Maternal fasting blood samples were obtained by venipuncture during each trimester. Serum samples were immediately frozen in aliquots and stored at -80 C. ARA, EPA, and DHA were analyzed by gas chromatography–mass spectrometry. Lipids were extracted with 4 mL of chloroform and methanol (1:1) from serum (200 μl) containing 25 μL of internal standard (fatty acid 17:0 as phospholipid, 1 mg 17:0/mL); 2 mL of 0.5% NaCl solution was added, and the chloroform phase was collected [21]. The water phase was washed with 2 mL of chloroform, and the collected chloroform was evaporated and redissolved in 200 μL of chloroform. Phospholipids were separated on an aminopropyl solid- phase extraction column [22], evaporated, dissolved in 1 mL of toluene, converted to methylesters by direct trans-esterification [23], extracted with petroleum ether, and evaporated again. The phospholipids were dissolved in isooctane separated by gas chromatography, and detected by mass spectrometry. Chemstation software (Agilent Technologies, Santa Clara, CA) was used for evaluation. The samples were separated by gas chromatography on a VF-WAX (30 m × 0.25 × 0.25 μm d_F_) column (J & W Scientific, Folsom, CA) and quantified by electron ionization with a 5975C inert XL EI/CI MSD with a triple-axis detector (Agilent Technologies). The coefficient of variation of eight separate extractions and quantifications of one plasma sample was 1.8% for s-ARA, 2.9% for s-EPA, and 3.4% for s-DHA.

### Statistical analyses

SPSS version 21 (SPSS, Chicago, IL) was used for statistical analyses. Results of parametric tests are presented as mean (SD) and results of nonparametric tests as median with interquartile range (25^th^ percentile, 75^th^ percentile). Sample sizes were calculated in two ways. In the intervention group, six women were needed to detect an increase in fish intake of 50 g/week (SD 20 g) between the first and third trimesters. Furthermore, five women were needed in each group to detect a difference in fish intake of 50 g/week (SD 20 g) between control and intervention groups in the third trimester. These power calculations were based on Students t-test, with a significance level of 5% and a power of 90%. The reported fish and meat intake was checked for outliers. One outlier was found for fish intake, and two outliers were found for meat intake. Since these were judged to be true estimates of intake, they were included in the analyses. Nonparametric analyses for repeated measures (i.e., Friedman test followed by post-hoc Wilcoxon signed-rank tests) were used for subgroup analyses of women (the subgroup analysis was performed ad-hoc) who had complete measurements of body composition, fish intake, and serum phospholipid fatty acids in all three trimesters. Normality was checked graphically and non-parametric tests were performed due to low numbers of women. Correlations were done by the Spearman rank order correlations between the following variables: fish/meat intake and serum phospholipid fatty acids (ARA, EPA, and DHA), GWG, FM gain, FFM gain, birth weight, and length at birth. The Mann–Whitney U test was used to compare cross-sectional data from two groups. Fisher’s exact probability test was used to check for differences in number of women in the intervention and control groups who reached the recommended fish intake. For some analyses, data from women in the first trimester (“early pregnancy”) were pooled to a maximum large baseline population. Thus, all women included in this analysis were analyzed at baseline, regardless of any missing data or later drop-out. The “early pregnancy” data were analyzed with parametric tests (i.e. Student’s independent- samples *t*-test and Pearson’s correlation test).

## Results

### Study population

Initially, 101 women agreed to participate in the study and 49 were randomized to interventions and 52 to controls (Figure 2). Seven women were excluded and there were 13 drop-outs after their first study visit and additionally one after trimester 2. Data from 73 women were pooled and analyzed at baseline (“early pregnancy”), whereas 35 women had complete measurements from all trimesters (Figure 2). Mann–Whitney U tests showed no differences in age, parity, or BMI between the drop-outs and women who completed the study (data not shown).

### Subjects at baseline

The average age of the participants in the early pregnancy population was close to 31 years (Table 1), and 56% of these women were primiparous. According to self-reports, none of the women smoked during the first trimester (data not shown). The education level was high among the participants (Table 1 + Table 2). In the subgroup that had participated in all trimesters, 60% were primiparous.

**Table 1:** **Early pregnancy: characteristics at baseline** ^**1**^

	Control group ^2^	Intervention group ^3^	P ^7^
Age (years)	31.2 (4.0)	31.4 (3.9)	0.84
Weight (kg)	63.8 (6.4)	61.5 (5.8)	0.12
Height (cm)	170 (6.7)	167 (6.3)	0.08
Body mass index (kg/m^2^)	22.0 (1.3)	22.0 (1.6)	0.88
Waist circumference (cm)	79.4 (4.8)	80.4 (6.6)	0.48
Fat mass (kg)	16.8 (3.5)	16.3 (4.3)	0.60
Fat-free mass (kg)	47.0 (4.8)	45.2 (4.6)	0.11
Parity (n)^4^	0 (0, 1)	0 (0, 1)	0.46^6^
15 or more years of education^5^	26 (72.2)	28 (75.7)	
Plasma glucose (mmol/L)	4.4 (0.4)	4.4 (0.4)	0.90
Serum insulin (mU/L)	5.0 (2.5)	5.1 (3.4)	0.88
QUICKI^8^	0.340 (0.050)	0.337 (0.044)	0.81

^1^Mean (SD) unless noted otherwise. ^2^n = 35-36. ^3^n = 34-37. ^4^Median with interquartile range (25^th^ percentile, 75^th^ percentile). ^5^n (%). ^6^Analyzed by Mann–Whitney U test. ^7^P values were calculated with Student’s independent-sample *t* test. ^8^Quantitative insulin sensitivity check index.

**Table 2:** **Subgroups: characteristics at baseline of women who participated in all three trimesters** ^**1**^

	Control group ^2^	Intervention group ^3^	P ^5^
Age (years)	30.6 (29.0, 32.5)	32.2 (30.3, 33.3)	0.19
Weight (kg)	63.7 (60.1, 68.0)	61.9 (59.0, 65.1)	0.32
Height (cm)	173 (167, 178)	166 (162, 173)	0.05
Body mass index (kg/m^2^)	22.3 (20.9, 22.7)	22.3 (20.9, 23.3)	0.62
Waist circumference (cm)	80.0 (77.5, 82.5)	81.9 (78.0, 86.0)	0.26
Fat mass (kg)	16.0 (14.5, 18.9)	16.6 (12.1, 22.1)	0.90
Fat-free mass (kg)	47.3 (44.7, 52.9)	45.6 (41.5, 49.1)	0.15
Parity (n)	0 (0, 1)	0.5 (0, 1)	0.26
15 or more years of education^4^	15 (88.2)	17 (94.4)	
Plasma glucose (mmol/L)	4.3 (4.3, 4.8)	4.3 (4.3, 4.5)	0.83
Serum insulin (mU/L)	4.9 (4.3, 5.6)	4.6 (4.0, 5.6)	0.70
QUICKI^6^	0.323 (0.307, 0.340)	0.338 (0.312, 0.352)	0.47

^1^Median with interquartile range (25^th^ percentile, 75^th^ percentile) unless noted otherwise. ^2^n = 17. ^3^n = 17-18. ^4^n (%). ^5^P values were calculated by Mann–Whitney U test. ^6^Quantitative insulin sensitivity check index.

### Early pregnancy

To better understand the effects of fish and meat intake alone on serum phospholipid fatty acids and body composition, we pooled first trimester data from the two groups for some analyses. Seventy-seven percent reached the recommendation of three servings of fish per week. Mean intakes were as follows: fish 384 g/week (210), n = 69; meat 1112 g/week (525), n = 69; energy 2234 kcal/day (543), n = 69. Mean serum fatty acid concentrations were as follows: ARA: 0.200 mg/mL (0.044), n = 73; EPA: 0.029 mg/mL (0.014), n = 73; DHA: 0.142 mg/mL (0.043), n = 73. Serum EPA (r = 0.36, p = 0.002, n = 69) and s-DHA (r = 0.34, p = 0.005, n = 69) correlated positively with fish intake reported in the first trimester, but no significant correlations were found with meat intake (data not shown). Serum ARA correlated positively with meat intake in the first trimester (r = 0.28, p = 0.02, n = 69), but no significant correlation was found with fish intake (data not shown). GWG nearly achieved a significant positive correlation with reported meat intake in the first trimester (r = 0.35, p = 0.053, n = 45). Also, FFM gain correlated positively with meat intake in the first trimester (r = 0.39, p = 0.009, n = 45). Fish intake in the first trimester did not correlate significantly with either GWG or FFM gain (data not shown). FM gain did not correlate with fish intake or meat intake in the first trimester. No significant correlations were found between baseline meat intake and fish intake and either weight or length at birth. In the baseline population, three women used supplements containing fatty acids.

### Reported fish intake during pregnancy

Reported baseline fish intake was lower in the intervention group than in the control group (Table 3), but the difference was not significant. Fish intake did not differ between the groups in any of the trimesters. The intervention group significantly increased their fish intake from the first to the second trimester and from the first to the third trimester.

**Table 3:** **Reported intake of fish, meat, and energy in all three trimesters** ^**1**^

	Fish intake (g/week)			Meat intake (g/week)			Energy intake (kcal/day)		
Trimester	Control group^2^	Intervention group^3^	P^5^	Control group^2^	Intervention group^3^	P^5^	Control group^2^	Intervention group^3^	P^5^
1^st^	450 (300, 600)	300 (150, 450)	0.10	1050 (788, 1750)	963 (525, 1269)	0.26	2282 (1992, 2936)	2161 (1814, 2335)	0.25
2^nd^	450 (300, 600)	413 (225, 488)^6^	0.78	1225 (700, 1750)	1006 (678, 1312)	0.29	2419 (2026, 2878)	2196 (2008, 2510)	0.44
3^rd^	450 (300, 525)	375 (300, 600)^7^	0.88	1400 (700, 1925)	1007 (875, 1269)	0.21	2330 (2010, 2678)	2364 (2033, 2860)	0.72
P^4^	0.98	0.006		0.49	0.35		0.44	0.66	

^1^Values are medians with interquartile range (25^th^ percentile, 75^th^ percentile). ^2^n = 15-17. ^3^n = 18. ^4^Friedman test analyses within treatment groups, followed by post-hoc Wilcoxon signed-rank tests. ^5^Differences between groups in each trimester were analyzed by Mann–Whitney U tests. ^6^P = 0.03 between the first and second trimesters. ^7^P = 0.01 between the first and third trimesters.

### Reported meat intake during pregnancy

Meat intake was lower in the intervention group than in the control group in all three trimesters, but the difference was not statistically significant (Table 3). In both groups, meat intake increased from the first to the third trimester, but the increase was not statistically significant (Table 3).

### Reported energy intake during pregnancy

Energy intake did not differ between the groups in any of the trimesters or within the groups between trimesters (Table 3). However, reported energy intake showed a non significant trend to increase in the intervention group in the third trimester.

### Serum phospholipid fatty acids during pregnancy

The median concentration of ARA increased significantly from the first to the second and from the first to the third trimester in both groups, and also from the second to the third trimester in the intervention group (Table 4). The s-DHA increased in both groups from the first to the second and from the first to the third trimester (Table 4). Serum EPA levels increased from the first trimester to the third trimester in the intervention group (ns), and was higher than the control group in the third trimester, but the differences were not significant. Levels of s-DHA, s-EPA and s-ARA did not differ significantly between the groups in any of the trimesters (Table 4).

**Table 4:** **Serum concentrations of ARA (arachidonic acid) (mg/mL), EPA (eicosapentaenoic acid) (mg/mL), and DHA (docosahexaenoic acid) (mg/mL) in all three trimesters** ^**1**^

	ARA (mg/mL)			EPA (mg/mL)			DHA (mg/mL)		
Trimester	Control group^2^	Intervention group^3^	P^5^	Control group^2^	Intervention group^3^	P^5^	Control group^2^	Intervention group^3^	P^5^
1^st^	0.185	0.193		0.027	0.030		0.132	0.154	
	(0.153, 0.222)	(0.167, 0.232)	0.46	(0.017, 0.040)	(0.018, 0.043)	0.76	(0.110, 0.157)	(0.115, 0.174)	0.37
2^nd^	0.202	0.216		0.027	0.035		0.162	0.189	
	(0.187, 0.240)^6^	(0.174, 0.235)^9^	0.83	(0.019, 0.051)	(0.020, 0.045)	0.66	(0.137, 0.203)^12^	(0.159, 0.261)^13^	0.12
3^rd^	0.227	0.231		0.024	0.042		0.196	0.197	
	(0.189, 0.256)^7^	(0.195, 0.270)^8,10^	0.64	(0.018, 0.040)	(0.019, 0.066)	0.16	(0.155, 0.217)^11^	(0.145, 0.267)^14^	0.48
P^4^	0.01	<0.001		0.63	0.12		<0.001	<0.001	

^1^Values are medians with interquartile range (25^th^ percentile, 75^th^ percentile). ^2^n = 17 (for all time points). ^3^n = 18 (for all time points). ^4^Friedman test within each treatment group, followed by post-hoc Wilcoxon signed-rank tests. ^5^Differences between groups in each trimester were analyzed by Mann–Whitney U tests.^6^P = 0.01 between first and second trimesters. ^7^P = 0.006 between first and third trimesters. ^8^P = 0.004 between first and third trimesters. ^9^P = 0.006 between first and second trimesters. ^10^P = 0.02 between second and third trimesters. ^11^P < 0.001 between first and third trimesters. ^12^P = 0.002 between first and second trimesters. ^13^P = 0.003 between first and second trimesters. ^14^P = 0.003 between second and third trimesters.

### Fish and meat intake and serum phospholipid fatty acids

The s-EPA concentration correlated positively with reported fish intake during the first trimester in both groups (Table 5). Serum DHA correlated positively with fish intake in the first trimester in the intervention group, and in the second trimester in the control group. Meat intake was negatively correlated with s-EPA in the control group in the second trimester (Table 5). Serum ARA did not correlate with the meat intake in any of the trimesters.

**Table 5:** **Spearman’s rank correlations between fish intake (grams/week), meat intake (grams/week), and serum concentrations of ARA (arachidonic acid) (mg/mL), EPA (eicosapentaenoic acid) (mg/mL), and DHA (docosahexaenoic acid) (mg/mL) in all three trimesters**

	Control group ^1,3^						Intervention group ^2^					
	ARA mg/mL	P	EPA mg/mL	P	DHA mg/mL	P	ARA mg/mL	P	EPA mg/mL	P	DHA mg/mL	P
Fish intake grams/week												
1^st^ trimester	0.09	0.73	0.50*	0.04	0.46	0.07	0.16	0.53	0.78*	<0.001	0.69*	0.002
2^nd^ trimester	0.42	0.10	0.33	0.20	0.56*	0.02	-0.12	0.64	0.07	0.79	0.06	0.82
3^rd^ trimester	0.02	0.94	0.00	0.99	0.15	0.58	0.08	0.75	0.45	0.06	0.22	0.39
Meat intake grams/week												
1^st^ trimester	0.26	0.31	-0.06	0.82	0.11	0.66	0.37	0.13	-0.08	0.76	0.37	0.14
2^nd^ trimester	0.02	0.95	-0.59*	0.02	-0.28	0.31	0.19	0.46	0.06	0.82	0.21	0.40
3^rd^ trimester	0.04	0.88	-0.04	0.89	-0.02	0.95	-0.03	0.91	0.19	0.45	0.23	0.36

^1^n = 17. ^2^n = 18. ^3^n = 15 for meat intake in the second trimester. P values were calculated by Spearman’s rank correlation. *P<0.05.

### Gestational weight gain and body composition during pregnancy

The gains in FM and FFM from the first to the third trimester did not differ significantly between the two groups (Table 6). In neither group did GWG or gains in FM or FFM correlate significantly with fish or meat intake between the first and third trimester (data not shown).

**Table 6:** **Gestational weight gain (kg) and body composition changes (kg) between the first and third trimesters** ^**1**^

	Control group ^2^	Intervention group ^3^	P ^4^
Gestational weight gain (kg)	11.6 (9.5, 11.6)	11.6 (8.9, 13.9)	0.96
Fat mass gain (kg)	5.5 (4.0, 6.6)	6.8 (3.8, 8.4)	0.34
Fat-free mass gain (kg)	6.7 (4.9, 8.1)	5.5 (4.5, 6.7)	0.22

^1^Values are medians with interquartile range (25^th^ percentile, 75^th^ percentile), kg. ^2^n = 17. ^3^n = 18. ^4^P values were calculated by Mann–Whitney U test.

### Supplements with fatty acids

Women in the intervention group did not use supplements containing fish oil or n-3 fatty acids during pregnancy. In the control group, such supplements were used by one woman (6%) in the first trimester, two women (12%) in the second, and four women (24%) in the third. Serum levels of DHA and EPA are shown in Table 7.

**Table 7:** **Levels of serum EPA (eicosapentaenoic acid) (mg/mL) and DHA (docosahexaenoic acid) (mg/mL) in users and nonusers of supplements with fatty acids** ^**1**^

	Supplement users	Number of observations	Nonuser of supplements	Number of observations
S-EPA				
First trimester	0.054 (0.054, 0.054)	1	0.027 (0.017, 0.037)	34
Second trimester	0.071 (0.051, 0.091)	2	0.032 (0.020, 0.039)	33
Third trimester	0.032 (0.031, 0.059)	4	0.029 (0.016, 0.051)	31
S-DHA				
First trimester	0.157 (0.157, 0.157)	1	0.138 (0.113, 0.164)	34
Second trimester	0.216 (0.196, 0.235)	2	0.177 (0.151, 0.222)	33
Third trimester	0.209 (0.187, 0.219)	4	0.196 (0.150, 0.237)	31

Serum levels in women with measurements in all three trimesters.^1^Values are medians with interquartile range (25^th^ percentile, 75^th^ percentile), mg/mL.

### Birth weight and length

There were no significant differences in birth weight or birth length between the two groups (data not shown). Fish intake and meat intake in the second and the third trimesters did not correlate with birth weight or birth length in any of the groups. Similarly, GWG, FM gain, and FFM gain did not correlate with birth weight or birth length (data not shown).

## Discussion

This study shows that a longitudinal dietary intervention can increase fish intake in pregnant women of normal weight, and that meat intake during early pregnancy may be associated with FFM gain. Additionally, serum phospholipid EPA and DHA correlated with fish intake and ARA correlated with meat intake in early pregnancy.

### Fish, meat, and energy intake during pregnancy

A high percentage of the women reported a satisfactory fish intake at baseline (384 g/week, or 55 g/day), which was higher than the 33 g/day among women aged 31–44 years in a Swedish national survey [24]. The control group did not increase their intake of fish significantly during pregnancy as the intervention group did, but then the intervention group also reported a (nonsignificantly) lower intake at baseline than the control women. The increased fish intake in the intervention group could reflect the success of the individualized counseling. However, self-reported intake could be biased, as the women in the intervention group might be more likely to report the recommended fish intake, without actually changing their diet. Likewise, all women that participated in the study might have over-reported their fish consumption if they considered fish to be a healthy food. Intake of socially desirable food groups leads to report bias [25], and pregnant women have over-reported their energy intake in accordance with dietary advice [26]. Thus, the high fish intake reported by the control group might have been influenced both by the social trends of eating fish and by the usual recommendations at the Swedish maternal health care center. However, this should influence the intervention group equally. In a Finnish study in which the intervention group received advice based on NNR 1996, the groups did not differ in their daily intake of meat or fish. The mean meat intake seemed to be lower in the intervention group than in the control group in the third trimester (no statistics were reported on this) [27].

There is therefore no evidence that women automatically increase their fish intake during pregnancy unless advised to do so. Not only fish intake was lower in the intervention group than in the control group; meat and energy intake were also lower in both the first and the second trimesters; however, the differences were not statistically significant. In the intervention group, energy intake increased by ~200 kcal per day from the first to third trimester. Although this increase was not statistically significant, it indicates that the intervention group followed the recommendations given by the dietary intervention.

### Serum phospholipid fatty acids

We wanted to compare possible dietary changes by using biomarkers for fish intake, such as s-EPA and s-DHA. Both correlated with fish intake in the first trimester and s-DHA correlated with fish intake in one of the groups in the second trimester. The reported increase in fish intake in the intervention group could not be confirmed by correlations with s-EPA and s-DHA in the second and the third trimesters but was confirmed by an increase in s-DHA. In nonpregnant conditions, the fatty acids in blood are extensively used biomarkers for fatty acid intake [28], as these only to a limited extent are endogenously synthesized from α-linolenic acid [29]. Fat metabolism is altered during pregnancy; initially fat is stored in the fat depots, but later in pregnancy breakdown of fat tissue [30], leads to higher levels of free fatty acids in the blood [31]. Pregnancy itself affects the fatty acid profiles of the mother, owing to the natural fat deposition that occurs during this period [32, 33]. Plasma phospholipid concentrations increase during pregnancy [34], and there is an active transport of PUFAs, particularly DHA, across the placenta to the fetus [35]. Also, it might be possible that EPA and DHA are consumed during pregnancy for production of eicosanoid-derived mediators like prostaglandins. We have earlier observed such consumption during inflammatory states when prostaglandin production is needed [36, 37].

Fish intake in the intervention group increased from the first trimester to both the second and third trimesters. Yet, the increase in fish intake could not be verified by positive correlations with the serum fatty acid levels, possibly because of uptake by the fetus or dilution in the increased blood volume. Therefore, new tools and biomarkers should be identified that could help support reported food intakes in pregnant women. Notably, median s-EPA in the third trimester was higher in the intervention group than in controls, although the difference was not statistically significant. However, fish intake did not differ between the groups in the third trimester. Thus, the higher s-EPA in the intervention group might reflect contributions from other food sources such as seaweed products (not analyzed in this report), or supplements. The correlation between the reported fish intake and s-EPA and s-DHA in early pregnancy confirms that the women’s reported fish intake was sufficient, and these fatty acids have previously been used as biomarkers for fish intake in early pregnancy, although they were measured in erythrocytes [38] and not in serum phospholipids as in the present study.

Throughout pregnancy, serum EPA and s-DHA were lower in the control group than in the intervention group, but not significantly. This discrepancy between their higher reported fish intake might reflect over-reporting. However, there is no reason to expect greater over-reporting by controls than by women in the intervention group, and one can assume large interindividual variations in over-reporting. Fatty fish and lean fish were not separated in this present analysis. However, reported intake and plasma phospholipid DHA and EPA correlated strongly with consumption of both fatty and lean fish [39]. Thus, intake of fatty or lean fish should not be a source of error for the lack of correlation between s-DHA and s-EPA and reported fish intake.

The correlation in early pregnancy between s-ARA in the analyses of the larger baseline group and the meat intake in the first trimester also validates the reported meat intake. The lack of correlations in the subgroup could reflect the smaller number of subjects. There are also other dietary sources of ARA that we did not study, such as eggs.

The proportion of PUFAs in the blood has been found to decrease between the first and the third trimesters [40]. The total amount of plasma DHA was reported to increase and then stabilize in later pregnancy, and plasma ARA was reported to increase throughout pregnancy [41], as it did in our study. A larger study failed to find any correlation between s-DHA and fish intake in the third trimester [42]. The authors attributed that finding to large physiological changes during pregnancy and concluded that even in a population with a high fish intake, DHA levels decrease in the later stage of pregnancy because of increased DHA demands of the fetus. However, another study did find positive correlations between erythrocyte DHA and fish intake in later pregnancy (gestational week 36) [43]. Biomarkers can be difficult to use as they reflect both endogenous and exogenous factors [44], especially during pregnancy. The observed differences in correlations between blood DHA and fish intake could reflect analysis of different fractions of the blood. We analyzed phospholipid fatty acids in lipoproteins; thus, we did not measure fatty acids in all blood fractions, such as erythrocytes and serum triglycerides. We also focused on absolute amounts of the fatty acids of interest and therefore we did not check for changes in the relative composition of the phospholipid fraction (i.e. whether there was any difference in the levels of EPA, DHA, and ARA as a percentage of the total fatty acids). Fatty acids in serum and plasma may mirror the dietary fatty acid intake during the preceding weeks, whereas the fatty acids in erythrocytes may reflect intake during the preceding months [45].

### Gestational weight gain and body composition changes

In the baseline population, GWG correlated with borderline significance with meat intake in the first trimester. Few previous studies have investigated how GWG is associated with different foods or food consumption patterns; only one study reported meat intake [46], and none reported fish intake. The “fast food” pattern was positively associated with GWG rate in a Finnish population [47]. Another study examined determinants of excessive GWG, but food and food groups were not independently associated with excessive GWG [48]. On the contrary, intake of dairy and fried foods was associated with excessive GWG, whereas intake of “red and processed meat” was not higher among women with excessive GWG [46]. Furthermore, intake of proteins and fats of animal origin in the second trimester were positively linked to GWG [49]. In that study, however, no distinction was made between proteins and fats from different animal origins. Our study indicates that there might be reasons to distinguish between these nutrients from different animal sources. Furthermore, we found that the gain in FFM was correlated with meat intake in early pregnancy. No correlations were found between GWG or body composition changes and birth weight.

Possible connections between maternal body composition or body composition changes during pregnancy and birth weight have previously been investigated. FM gain was not correlated with birth weight [50, 51]; however, birth weight was associated with FFM gain [50]. Furthermore, maternal FM in term pregnancy was not associated with birth weight [52]. These findings show the importance of separating weight into FM and FFM components. To our knowledge, previous studies have not examined the associations between fish intake and meat intake and changes in maternal body composition during pregnancy. Other possible determinants of body composition during pregnancy in normal weight women include physical activity, the rate of edema, or consumption of foods that we did not analyze.

Average GWG in our normal weight women was slightly below the Institute of Medicine 2009 recommendations [4], and also less than in Swedish women of normal weight [2]. The present GWG was calculated as the difference between measured weight at the study visits during pregnancy weeks 8–12 and 35–37. Thus, weight was not measured before conception or during the last 3–5 weeks of pregnancy, and could have introduced an error in the GWG assessment. However, in studies of women with average normal BMI, the average GWG in the first trimester determined by measurement of weight before conception and in the first trimester was 0.2 - 1.8 kg [50, 53, 54]. Therefore, although GWG might be slightly biased compared to other studies, such a bias would not affect our comparisons within this study.

The average FM gain was more than in “well-nourished women” in gestational week 36 [1]. The present high FM gain in normal weight women could truly reflect a high deposition of fat. However, air-displacement plethysmography uses the two-compartment model based on densitometry [55], with an assumed FFM density of 1.1000 kg/L. About 6–8 kg of total body water accumulates during pregnancy [50, 53, 54], and hydration of FFM in late pregnancy is 74-76% [53, 54, 56, 57]. Consequently, the density of FFM in late pregnancy is affected and reported to be 1.087-1.089 kg/L [56–58], violating the assumed fixed density of FFM. Therefore, the decreased density of FFM in pregnancy could result in overestimation of FM [55]. Additionally, predicted, and not measured, thoracic gas volume was used in this study. However, one study [59] concluded that, although biased, predicted thoracic gas volume was suitable for pregnant women in most cases. Thus, predicted thoracic gas volume is probably not a considerable source of error.

### Birth weight and length in relation to fish and meat intake

In some studies, high intake of fish or seafood was associated with higher birth weights [60–62]. We found no such associations in the present study. However, our sample size was smaller than in these studies and might not have sufficient power to find correlations between these variables. Also, we did not find any differences between birth weights of newborns in the intervention and the control groups; however, this was not one of the aims of the intervention.

A weakness of this study is that we focused solely on fish and meat intake. Nonpregnant women with a high intake of fish (especially oily fish) also have a higher reported intake of dietary supplements and consume more fruits and vegetables than meat eaters and vegetarians [63]. Selection bias is a possibility in our study, as the participants already had a high consumption of fish, perhaps because women who were more health conscious agreed to participate in the study. A higher education level, as in our participants, is linked to healthy food choices. Additionally, the size of the study population was rather small, which may have reduced the power to find differences or correlations. Also, errors may have been introduced by the use of a food frequency questionnaire that has not been validated and that uses standard portions sizes.

A strength of our study was the enrolment of women early during pregnancy. However, this also resulted in drop-outs and exclusions due to miscarriages, abortions, and multiple fetuses/births, thereby reducing the precalculated statistical power of the study. Another strength is the longitudinal design allowing repeated measurements of the same variables at three times during pregnancy. Instead of only looking at weight gain in the women, we also measured body composition using a method that is safe both for the woman and the fetus. Our study shows that dietary interventions work and illustrates the importance of an active dialogue about nutrition between health professionals and pregnant women. This approach would require that health personnel have time to do structured nutritional counseling, including both personal visits and telephone calls during pregnancy. Fish intake during pregnancy has been decreasing in the US after authorities started to warn pregnant women about environmental toxins in fish [64]. However, as fish and shellfish are important contributors of fatty acids, vitamin D, proteins, and minerals, it is advisable that women of child-bearing age receive proper guidance to choose the right types of fish from nonpolluted waters. Awareness should be raised about the importance of a healthy diet throughout pregnancy.

## Conclusions

In conclusion, this study shows that dietary counseling during pregnancy can increase fish intake. Meat intake in the first trimester is positively associated with gain in FFM during pregnancy. In early pregnancy, serum phospholipid EPA and DHA are correlated with fish intake, whereas ARA is correlated with meat intake.

## Abbreviations

ARA: Arachidonic acid
BMI: Body mass index
BW: Body weight
DHA: Docosahexaenoic acid
EPA: Eicosapentaenoic acid
FFM: Fat-free mass
FM: Fat mass
GWG: Gestational weight gain
LC: Long chain
NNR: Nordic Nutrition Recommendations
PONCH: Pregnancy Obesity Nutrition and Child Health
PUFA: Polyunsaturated fatty acid
QUICKI: Quantitative insulin sensitivity check index.
